# Development and Psychometric Validation of the Career Identity Questionnaire for Vocational School Students

**DOI:** 10.3390/bs16050834

**Published:** 2026-05-21

**Authors:** Branimir Vukčević, Teodora Safiye, Elvis Mahmutović, Emir Biševac, Nemanja Nenezić, Andreja Kovačević, Aleksandar Stevanović, Zerina Salihagić, Aldina Ajdinović, Velida Zimonjić, Aleksandar Jeremić, Ernad Kahrović, Zana Dolićanin, Draško Dubljanin

**Affiliations:** 1 Hospitality and Tourism School with Dormitory, 36210 Vrnjacka Banja, Serbia; psiholog@uts.edu.rs; 2 Department of Psychology, State University of Novi Pazar, 36300 Novi Pazar, Serbia; 3 Department of Biomedical Sciences, State University of Novi Pazar, 36300 Novi Pazar, Serbia; ehmahmutovic@np.ac.rs (E.M.); ebisevac@np.ac.rs (E.B.); zsalihagic@np.ac.rs (Z.S.); aajdinovic@np.ac.rs (A.A.); zdolicanin@np.ac.rs (Z.D.); 4 Department of Medical Studies Ćuprija, Academy of Educational and Medical Vocational Studies Kruševac, 35230 Cuprija, Serbia; nenezicn@avmss.edu.rs; 5 Faculty of Medical Sciences, University of Kragujevac, 34000 Kragujevac, Serbia; doktorkole86@gmail.com (A.K.); draskodubljanin@gmail.com (D.D.); 6 Department of Cardiopulmonary Rehabilitation, Institute for Rehabilitation Belgrade, 11000 Belgrade, Serbia; 7 International Doctoral School, Catholic University of Murcia, 30107 Murcia, Spain; aleksandarstevanovic@vsovsu.rs; 8 Quality Assurance Office, State University of Novi Pazar, 36300 Novi Pazar, Serbia; vkijevcanin@np.ac.rs; 9 Department of Pulmonology, University Clinical Hospital Center Zvezdara, 11120 Belgrade, Serbia; a.jeremic94@gmail.com; 10 Department of Economic Sciences, State University of Novi Pazar, 36300 Novi Pazar, Serbia; ekahrovic@np.ac.rs

**Keywords:** career identity, career exploration, career commitment, vocational education, questionnaire validation, adolescents, mental health

## Abstract

This study presents the development and psychometric validation of a Career Identity Questionnaire for vocational school students aged 15–19 years. Career identity was conceptualized as comprising career exploration and career commitment, based on theories of identity development and career guidance. Two studies were conducted. In Study 1 (*N* = 188), principal component analysis (PCA) was used to determine the final structure of the questionnaire. From an initial pool of 20 items, 14 items were retained: 8 items for career exploration and 6 items for career commitment. The two scales were positively correlated (r = 0.48, *p* < 0.01), with Cronbach’s alpha coefficients of 0.78 and 0.88, respectively. In Study 2 (*N* = 293), convergent and structural validity were examined using correlation analysis, canonical correlation, ANOVA, and confirmatory factor analysis (CFA). The CFA supported a three-factor structure consisting of exploration within the educational profile, exploration outside the educational profile, and career commitment. Career exploration correlated with school satisfaction (r = 0.31), subjective well-being (r = 0.26), and school success (r = 0.14), while career commitment showed stronger associations with the same variables (r = 0.56, 0.27, and 0.15, all *p* < 0.01). Canonical correlation analysis indicated significant relationships between career identity dimensions and mental health, with the strongest weights observed for career commitment (r_s_ = −0.96) and career exploration (r_s_ = −0.60), explaining 14% of the variance in mental health, while mental health explained 23% of the variance in career identity. Students with below-average values of commitment and exploration reported lower school success, satisfaction, and well-being. The findings provide evidence for the reliability and validity of the questionnaire, supporting its use in both research and practice in educational settings.

## 1. Introduction

An individual’s career identity is a key factor in education and work, and it is also one of the most important positive factors in mental health (Brown & Lent, 2021; Amundson et al., 2015). Although countless papers have been written on the development of the adolescent identity, until now, there has been no specific questionnaire concerning the career identity of vocational school students. The vocational school students in this context are those aged approximately 15–19 years, who attend a level of vocational education between primary school (aged 7–14) and college (aged 19 years or older). Students in vocational education develop their career identity through experiences within their chosen educational profile, as well as through broader school and extracurricular activities. Existing instruments allow for the assessment of overall levels of career commitment and exploration (Porfeli et al., 2011; Meeus et al., 2012; Crocetti, 2017), but do not distinguish whether these processes are directed toward the selected educational profile or toward alternative career pathways. Consequently, it remains unclear in which context career identity is primarily developed. To address this limitation, the present study introduces an instrument whose items explicitly reflect students’ experiences within vocational education. This approach enables a more precise differentiation between career commitment and exploration directed toward the educational profile and those oriented toward alternative career options. In addition to its theoretical contribution, the instrument has practical relevance, as it enables vocational schools to assess the extent to which students’ career commitment and exploration are aligned with their chosen vocational pathway, as opposed to being directed toward alternative options—an aspect not captured by existing measures. The instrument developed in this study enables the identification of students who may require additional support in career guidance and counseling in vocational school, which also represents its practical value.

In the twenty-first century, frequent job changes have made career guidance and counseling in schools a priority. Its main outcome is the development of students’ competencies for effective career planning and realization. Contemporary approaches build on the paradigm established by Frank Parsons as early as 1905 (Amundson et al., 2015), which emphasizes three key elements: understanding one’s abilities and interests, acquiring knowledge about occupations, and evaluating the relationship between the two. However, in modern labor markets, aligning interests and abilities with job characteristics is no longer sufficient. Individuals are also required to actively develop their personal potential in order to navigate career transitions and achieve long-term success. Accordingly, contemporary career guidance theories and practices have evolved from Parsons’ foundational framework (Brown & Lent, 2021; Amundson et al., 2015).

The study of vocational identity as part of personality development over an individual’s lifespan began with the research and theories of Erikson (1968) and Marcia (1980). During adolescence, an individual develops personal identity by successfully solving his or her own identity crisis. In short, a person’s identity is the answer to the questions of who I am and what I would like to be. Adolescents answer these questions by considering the knowledge, norms, values and other cultural and social assets of the community in which they live. Erikson and Marcia wrote about aspects of the self related to gender roles, careers, and political ideologies. Political ideology includes ethical ideals, principles, doctrines, symbols or myths of a social movement, institution, or other social group, which explain how society should function and what structure it should have. Difficulties in realizing oneself in the areas of career, gender role and political ideologies constitute an identity crisis in adolescence, whereas stable answers to the questions in those areas represent a solution to the personal identity crisis. Two processes in an individual’s personality are necessary to resolve a personal identity crisis. The first is called exploration, and the second is called commitment. Exploration refers to the active examination of the sociocultural environment to understand oneself and one’s life in that environment. Commitment refers to the stability of one’s understanding of oneself and the realization of one’s role in that sociocultural environment. A characteristic virtue of the individual’s ego that commitment requires is fidelity (Erikson, 1968). Acquiring that ego virtue is the goal of adolescence as a stage of development. The exploration that adolescents make during youth leads to their choices regarding their own behavior in relation to gender, work and political ideologies, whereas commitment implies fidelity to those choices. High exploration means less commitment, whereas more commitment means less exploration. It seems logical that when an adolescent intensively examines how suitable different social roles are for his personality, commitment in some social roles cannot be great. However, findings from later research show that this is not quite the case (Meeus et al., 2002, 2012; Crocetti et al., 2008; Crocetti & Meeus, 2014), which will be discussed later in this paper.

The theory of ego identity status includes successive phases of solving the crisis of personal identity (Erikson, 1968; Marcia, 1980). When the adolescent is just entering the identity crisis, the adolescent has weak exploration and weak commitment. This is the phase of identity diffusion. Under the pressures of adolescents’ own needs and sociocultural environment, their research begins to strengthen, but their commitment is still weak. This phase is called identity moratorium. After that, in the third phase, the pressure of the sociocultural environment overrides the adolescent’s needs and will, and the adolescent accepts forms of commitment that are not aligned with his or her needs. This is the phase of identity imposed by the social environment, which is called identity foreclosure, as if the social environment seized the adolescent’s personal identity and imposed on the adolescent the one that suits the social environment. The fourth and last phase implies a strong commitment that was born on the basis of free exploration, which gradually decreased after this period. Adolescents have found their own appropriate roles in the world, and the adolescents’ need for exploration is weakening. This phase is called identity achievement. Each mentioned phase represents one ego identity status. Research shows that there are phases of solving a personal identity crisis as theory predicts, but that the sequence of phases is not always the same. No one goes directly from identity diffusion to achievement. However, boys often enter the final phase, i.e., identity achievement from the moratorium, whereas girls enter it from the identity foreclosure phase, as the theory predicts (Meijers, 1998). In accordance with the gender role, compared with boys, girls pay more attention to social conventions, whereas the male gender role implies more freedom in making decisions and exploring reality outside the family and a friendly environment. Additionally, research shows that once the final stage is reached, this does not mean that it is the end of the development of ego identity status.

Building on Erikson’s theory and Parsons’ paradigm, Donald Super developed an influential theory of career development in which career choice is understood as an expression of one’s self-concept (Super, 1980; Super et al., 1996). Career development is viewed as a lifelong process encompassing the stages of growth, exploration, establishment, maintenance, and disengagement. Among these, the exploration stage is particularly significant, as it involves active examination of one’s interests, abilities, and opportunities within the world of work, leading to the gradual formation and crystallization of career identity. Effective career decision-making depends not only on knowledge of oneself, the world of work, and decision-making processes, but also on timely engagement in developmental tasks such as exploration and the narrowing of options. Super further conceptualizes career as a “Life–Career Rainbow,” emphasizing the interaction of multiple life roles across the lifespan (Brown & Lent, 2021; Amundson et al., 2015).

Extending Super’s framework, Savickas proposed career construction theory, highlighting the individual’s active role in constructing meaning in their career (Savickas, 2005). This approach includes three key components: vocational personality, life theme, and career adaptability. Exploration remains central within this perspective, as individuals, through narrative reflection on their experiences, identify patterns, values, and life themes that guide their career choices. Career adaptability refers to the competencies that enable individuals to cope with transitions and changes, facilitating the integration of self-concept into work roles. The counseling process, grounded in a narrative approach, supports individuals in linking their life stories with their career paths, thereby fostering a coherent and meaningful career identity (Wang & Li, 2024).

In Holland’s approach, career counseling focuses on a client’s work history and education as key indicators of interests and abilities (Holland, 1997). His theory is based on four main assumptions: personality can be described through six types—realistic, investigative, artistic, social, enterprising, and conventional; work environments reflect these same types; individuals are attracted to environments that match their personality; and congruence between person and environment leads to greater satisfaction and effectiveness. Accordingly, counseling emphasizes exploring the fit between personality and occupational environments (Brown & Lent, 2021; Amundson et al., 2015).

Krumboltz’s social learning theory of career development similarly focuses on career choices and decision-making (Amundson et al., 2015; Krumboltz, 1996). It highlights four key influences: genetic factors, environmental conditions, learning experiences, and task-approach skills. Career counseling aims to shape learning experiences, strengthen decision-making skills, and address irrational beliefs that may hinder development. Individual interpretations of experiences, shaped by reinforcement and role models, form beliefs and attitudes toward careers. Importantly, uncertainty is viewed as a positive condition, as it encourages exploration of new opportunities and supports ongoing career development (Brown & Lent, 2021; Amundson et al., 2015).

Across Super’s, Savickas’s, Holland’s, and Krumboltz’s frameworks (Brown & Lent, 2021; Amundson et al., 2015), career identity is consistently understood as developing through active exploration of personal characteristics in relation to opportunities and experiences in the world of work, with adolescence representing a particularly important period for this process. Despite theoretical differences, all approaches emphasize key domains of exploration—self-knowledge, environmental understanding, and experiential learning—as well as the dynamic interaction between the individual and context in shaping career identity. Accordingly, these famous approaches suggest that career identity assessment for students in vocational schools should be based on instruments that capture dimensions specific to their educational context. Such a questionnaire should include not only general domains of self-knowledge and career exploration, but also the particularities of vocational education, such as early contact with the world of work, practical training experiences, and the alignment between developing skills and occupational environments. In this way, assessment can more accurately reflect and support the formation of career identity within the specific educational and developmental context of these adolescents, while such an instrument would also have significant value as a tool in the process of career guidance and counseling in vocational schools.

Porfeli et al. (2011) developed and validated the Vocational Identity Status Assessment (VISA) as a multidimensional instrument intended to assess vocational identity across adolescents and young adults. The instrument integrates three key dimensions derived from contemporary identity models: career exploration (in-breadth and in-depth), commitment (commitment making and identification with commitment), and reconsideration (career self-doubt and flexibility), allowing for a comprehensive and context-general assessment of vocational identity development. Their findings support a six-status solution, including the four classical identity statuses (achieved, moratorium, foreclosed, and diffused), along with two additional statuses—searching moratorium and undifferentiated—indicating a more nuanced structure of vocational identity. The results further demonstrate that these dimensions and their configurations into identity statuses are systematically related to work valence and indicators of well-being. Specifically, higher levels of exploration and commitment, characteristic of the achieved status, are associated with more positive work valence, reflected in more favorable affective and experiential expectations regarding future work, as well as higher core self-evaluations and lower levels of depression, anxiety, and stress. In contrast, profiles marked by low commitment and exploration and elevated reconsideration, particularly career self-doubt, as seen in the diffused status, are linked to more negative work valence and poorer psychological adjustment. The searching moratorium status, despite relatively high exploration and commitment, is also characterized by elevated self-doubt and flexibility, which is associated with less favorable well-being outcomes compared to the achieved status. Other statuses, such as foreclosed and moratorium, exhibit intermediate patterns depending on the balance among exploration, commitment, and reconsideration. Overall, these findings indicate that vocational identity statuses, as configurations of underlying dimensions, play a significant role in shaping both work valence and broader indicators of psychological functioning (Porfeli et al., 2011).

Research by Šimunović et al. (2026) was conducted using the VISA instrument on a sample that included both gymnasium students and students from vocational schools. Their longitudinal findings showed that while gymnasium students exhibit more pronounced developmental changes in career commitment and exploration, vocational school students tend to display more stable identity profiles over time. This relative stability is likely related to earlier career choice processes among vocational students, whose identity formation is often consolidated prior to or at the very beginning of secondary education. Consequently, although the VISA dimensions capture key aspects of vocational identity in a general sense, they may be less sensitive to the specific developmental trajectories of students who have already entered a clearly defined professional pathway. These findings point to the importance of considering contextual specificities in vocational education and support the need for developing more specialized instruments that better reflect the characteristics of career identity formation among vocational school students.

### 1.1. Career Identity of Vocational School Students

To apply Erikson’s theory to the domain of career-related identity development, several key points of the theory must be considered. According to Erikson’s theory, personality develops in a predetermined order through eight stages of psychosocial development, from birth to death. During each stage, a person experiences a certain developmental crisis that can positively or negatively affect the development of personality and the health of the individual. The successful resolution of each crisis and the completion of each stage of development results in a healthy personality and the acquisition of basic virtues. The basic virtues are characteristic strengths that the ego can use to resolve later crises and throughout life in general. The epigenetic principle of Erikson’s theory implies the following: for the crisis of one developmental stage to be successfully resolved, the preceding developmental crisis must also be successfully resolved. Failure in the previous stage results in a reduced ability to complete further stages of development and, therefore, a less healthy personality and a less favorable self-concept (Erikson, 1968).

Commitment as the main factor of identity requires fidelity as a basic virtue of the ego, as Erikson wrote. Fidelity arises as a consequence of resolving the identity crisis. Fidelity implies the stability of ideas, feelings and motivations regarding career, gender and political ideologies. An adolescent must have the virtue of industry to successfully pass through an identity crisis. Industry is the virtue of ego which involves a sense of competence in the independent execution of tasks, and it is developed before adolescence, in middle and late childhood (Erikson, 1968). However, to acquire the virtue of diligence, an individual must first acquire the virtue of purpose. Virtue of purpose refers to the ability of independent initiative, which an individual should have acquired between the ages of three and six. To acquire the virtue of purpose, it is necessary for the individual to acquire the virtue of will, which implies the experience of independence in making decisions and governing oneself, which is acquired between the first and third years of life. The prerequisite for the virtue of will and the beginning of all virtues is found in the virtue of hope, which implies the experience of trust that arises in the first year of life. The trust that builds the virtue of hope arises in a child’s relationships with primary caregivers. Hope at the beginning of an individual’s psychic life represents the basis of the experience that the world is a place worth living in, which means being curious, exploring and developing oneself. The difficulties that adolescents face in developing their own career often stem from the weakness of some of the basic virtues of their ego. The weak virtue of industry makes it difficult to solve the identity crisis, which includes achieving fidelity within one’s career. When defining the concept and measuring career identity, one should consider the virtues of the ego that Erikson wrote about. The concept of a career should be defined beforehand.

In the theory of this paper, the notion of career is understood as it is done in the abovementioned modern theories (Amundson et al., 2015; Holland, 1997; Krumboltz, 1996; Savickas, 2002). An individual’s career is defined by all the activities and jobs in the world of work that the individual has performed in the past, is performing in the present, or will perform in the future, as well as his or her formal education and all other education that may relate to the world of work. The term “world of work” refers to all legal occupations, vocations or professions, and of course jobs, which a person does for a salary, fee and the like.

In accordance with the theories of Erikson (1968) and Marcia (1980), in this paper, we also define the career identity of vocational school students as part of the identity related to career. Thus, a student’s career identity includes career commitment and career exploration. The student’s career commitment is defined here as a preference for current schooling and vocational choice, which is defined by the student’s educational profile. In this research, the educational profiles of the Hospitality and tourism school in Vrnjačka Banja, Serbia, were cook, waiter, pastry chef, tourist technician, tourist–hotel technician, catering technician, hotel and restaurant technician, and culinary technician.

In the context of vocational education, commitment is not limited to a cognitive decision but also includes affective and experiential engagement with the chosen educational profile, reflected in students’ evaluation of vocational learning experiences. In the present study, this dimension is operationalized through items capturing students’ satisfaction with practical training, engagement with vocational subject content, and the relative preference for vocational compared to general education subjects. This conceptualization is consistent with the specific nature of vocational education, where identity development is closely tied to concrete learning experiences and early specialization within a chosen career pathway. Educational profile is a term that labels the type of school curriculum. In this context, the educational profile of a student is his or her vocational choice. A student’s career commitment includes experiences of satisfaction in learning school subjects that are directly related to the vocation, the experience of a better overlap of one’s own personality and one’s own education in a vocational school compared with other possible choices, belief in one’s own competence for education in a vocational school, and hope that the acquisition of vocational knowledge will be useful for a career in adulthood. Career exploration includes all activities by which a student expands knowledge and skills within the chosen vocational choice (i.e., educational profile), as well as learning about new opportunities for education or employment outside the chosen vocational choice.

Considering earlier scientific theories and research, it is reasonable to assume that the most favorable type of career identity in adolescence is the one that implies high commitment and high exploration. In this paper this career identity status is called career creating (CC). The most unfavorable career identity status is that which implies low commitment and low exploration. Here, it is called career stagnation (CS). Low commitment and high exploration represent a status that is named as career break (CB) because the students have a break in investing themselves in work or existing schooling to explore new possibilities in this regard. High commitment and low exploration represent a status called career rigidity (CR). The students rigidly stick to their own career path, and they are afraid of changes or new choices. Career identity status refers to the adaptation of adolescents in vocational education and the world of work and not to developmental stages, as was the case with identity status in the theories of Erikson (1968) and Marcia (1980). The child does not start the development of his career identity from the status of CS, because if it has healthy upbringing, speaking in the language of Erikson’s theory, the child possesses industry as an ego virtue upon entering puberty, which immediately allows the status of CC during vocational education. The definition of career identity in this paper is in accordance with Super’s theory, according to which adolescence is developmental stage of career exploration (Super, 1980; Super et al., 1996), so high career exploration throughout adolescence is normal and expected. Additionally, other well-known theories of career guidance and counseling indicate that the period of adolescence implies the exploration of career possibilities so that career commitment and career exploration grow or decline together (Amundson et al., 2015; Holland, 1997; Krumboltz, 1996; Savickas, 2002).

Considering the tight schedule of classes in contemporary schools, there is a need for a brief, economical, and reliable instrument that can be easily used to assess the career identity of vocational school students. The development of such an instrument emerged within a broader self-funded research project titled *Career Identity, Mental Health and Students’ Accomplishment*, led by Dr. Branimir Vukčević and Dr. Teodora Safiye. The project focuses on the development of assessment tools and career guidance interventions aimed at supporting the career development and well-being of vocational school students.

### 1.2. Career Identity, Mental Health, and School Satisfaction

The theories and practices of career guidance and counseling that are listed in this paper indicate that the mental health of individuals is intertwined with their good understanding of their own abilities and opportunities in the world of work (Amundson et al., 2015; Super et al., 1996; Holland, 1997; Krumboltz, 1996). In other words, a well-developed career identity is usually associated with good mental health.

Subjective well-being is understood as a positive factor in mental health. Earlier works have shown that, compared with adolescents with a less developed career identity, those with well-developed career identity have greater experiences of subjective well-being (Amundson et al., 2015; De Lise et al., 2024; Redekopp & Huston, 2018; Karas et al., 2015; Skorikov & Vondracek, 2011; De Goede et al., 1999; Krumboltz, 1993). The experience of career hope is associated with subjective well-being, which is a significant factor in the association of subjective well-being with career development (Shu et al., 2024). Career hope includes certainty in one’s own career goals, the ways to reach them, and the will to stay on that path despite difficulties or obstacles. Usually, those adolescents who believe that life is beautiful and meaningful also have the motivation to work on their own career.

Insurmountable difficulties or setbacks in the development of career identity in adolescents represent negative factors of their mental health. Adolescents’ symptoms of depression, anxiety, or distress may be intertwined with the difficulties they have in creating their careers (Kwon, 2024; Amaral et al., 2023; Dik et al., 2022; Marks et al., 2021; Hardin et al., 2006).

In addition to the aforementioned aspects of adolescent mental health, career development is associated with adolescent school engagement (Shu et al., 2024; Keijzer et al., 2022). The successful development of career identity among adolescents is associated with greater school engagement. School engagement, in addition to behavioral and cognitive engagement, also includes an emotional component related to satisfaction with school. In particular, the emotional component is connected with career identity; with greater satisfaction with school, career development is better. The satisfaction that adolescents have with their school can be treated as a positive factor in their mental health.

Despite the importance of career identity development during adolescence, there is still a lack of validated instruments specifically designed for vocational school students in Southeast Europe. Therefore, the aim of this study was to develop and validate the Career Identity Questionnaire for Vocational School Students and to examine its psychometric properties. To achieve this aim, two studies were conducted. The first study focused on the development of the questionnaire, while the second study examined its convergent validity.

## 2. Materials and Methods

### 2.1. Aim and Expectations of the First Study

The initial questionnaire was developed by Dr. Branimir Vukčević and Dr. Teodora Safiye and consisted of items formulated with consideration of the specific context of vocational education, which includes both general and vocational subjects, as well as the theoretical framework of career guidance (Brown & Lent, 2021; Amundson et al., 2015) and identity development (Erikson, 1968; Marcia, 1980). The development process was theory-driven and based on the domain-specific expertise of the authors. All items were formulated prior to data collection and were not modified based on exploratory results.

A two-factor structure was specified a priori, with the expectation that the instrument would comprise two dimensions: career commitment and career exploration. To assess career commitment in the context of vocational education, items were developed addressing students’ satisfaction with learning subjects directly linked to their future occupation, perceived alignment between personality and vocational education compared to other possible choices, beliefs regarding their competence within vocational schooling, and expectations that acquiring professional knowledge would be beneficial for their adult careers. Additionally, items were developed to capture career exploration, including activities through which students expand their knowledge and skills within their chosen career path (within the educational profile), as well as familiarity with alternative educational or occupational opportunities outside their primary vocational choice. In the first study, principal component analysis with Varimax rotation was applied solely as an exploratory method for assessing the underlying dimensional structure and reducing the initial item pool. This procedure was not used to generate the theoretical framework or to define the initial item content, but exclusively to evaluate whether the empirical data supported the hypothesized structure. The expectation was that the PCA results would support the theoretically assumed two-factor solution, resulting in two coherent subscales corresponding to career commitment and career exploration, respectively. Both subscales were expected to demonstrate satisfactory internal consistency.

### 2.2. Participants and Procedures in the First Study

To validate the construct measured by the career identity questionnaire, a survey was conducted on a random sample of 188 students, aged 15–19 (M = 16.55 ± 1.14). The students attended Hospitality and Tourism School with a student dormitory in Vrnjačka Banja, Serbia. The sample size was adjusted to the statistical condition that for factor analysis, there should be 5 to 10 times more respondents than questionnaire items when the items are expected to have factor saturations of approximately 0.5 and when a small number of factors are expected (Field, 2024; Flora & Flake, 2017).

The survey was conducted during October 2024 through the internet application Google Forms. The study procedures adhered to ethical standards in line with the Declaration of Helsinki. Participation was voluntary and anonymous, and all participants were informed of their right to withdraw at any time without consequences. Written informed consent was obtained from all participants, and for those under 18 years of age, consent was additionally obtained from their parents or legal guardians prior to participation. All data were kept confidential and accessible only to the research team. Ethical approval for the study was obtained from the Professional Ethics Committee of the State University of Novi Pazar, Serbia (approval number: 3059/24; 28 August 2024) and the Council of the Hospitality and Tourism School with Students’ Dormitory in Vrnjačka Banja, Serbia (approval number: 07–158; 13 September 2023).

### 2.3. Measures in the First Study

Ten items that were intended to form the career commitment scale and ten items that were designed to relate to the career exploration scale were created so that the initial version of the career identity status questionnaire consisted of twenty items. The items are statements, and the respondent is asked to rate how much he or she agrees that each statement applies to him or her on a five-point Likert scale, where 1 means “strongly disagree” and 5 means “strongly agree.” The scale of career commitment is operationalized via items related to (a) a preference for learning vocational school subjects of one’s educational profile in relation to general school subjects; (b) self-perception as a competent person for acquiring knowledge and skills within the vocation to which the educational profile that the student attends refers; and (c) the belief that one’s own choice of education for occupations that includes the chosen educational profile is appropriate for one’s own vision of oneself in the future. For example, in this school, some vocational subjects are serving food and drinks, cooking with practical lessons, the basics of tourism, the basics of catering and the basics of the economy of tourist companies. For example, in this school, some general subjects are mathematics, the Serbian language and literature, biology, chemistry, history and geography. The scale of career exploration was operationally defined using items related to: (1) researching the possibility of acquiring new knowledge and skills within the profession, which includes the chosen educational profile, and (2) acquiring new knowledge about occupations that are not covered by the chosen educational profile. The items of the initial version of the questionnaire were arranged so that the odd items refer to commitment, while the even items refer to exploration.

### 2.4. Data Analyses in the First Study

Descriptive measures were used for each item: minimum, maximum, mean value, standard deviation, skewness and kurtosis. A factor analysis was carried out, where the analysis of principal components was used as an extraction method. For the rotation method, varimax rotation was used. The KMO test of sampling adequacy (Kaiser–Meyer–Olkin measure of sampling adequacy) and Bartlett’s test of sphericity were used. The plan was to create scales for the questionnaire on the basis of the obtained factors that are interpretable in accordance with the theory of career identity. The procedure implied that the scale was formed from items with the highest saturation of the common factor. If the item has saturation with several factors, it is not added to the scale to avoid a high correlation between future scales and to reduce the size of the future questionnaire, thus making it more economical. A value of 0.3 was taken as the smallest acceptable value of item saturation with a common factor, whereas smaller values were not taken into account, which is usual in such analyses (Flora & Flake, 2017). The reliability of the scales was checked via internal consistency, i.e., Cronbach’s alpha. The IBM SPSS 25 program was used.

Principal Component Analysis (PCA) with Varimax rotation was applied to reduce the number of items, as it is consistent with the theoretical framework proposed by Marcia (1980), in which exploration and commitment are conceptualized as independent dimensions. This allows for a meaningful classification of participants based on the obtained results. The sample size was considered adequate for this procedure. A sample of 188 participants for 20 items represents an acceptable subject-to-item ratio for PCA, allowing for stable and interpretable solutions. Under such conditions, PCA can yield robust and replicable results, especially in the early stages of scale development. Finally, PCA maximizes the proportion of total variance explained, which contributes to greater internal consistency of the resulting subscales (Hair et al., 2022; Field, 2024).

### 2.5. Aims and Expectations of the Second Study

The first aim of the second study was to examine whether the constructs measured by the scales of the Career Identity Questionnaire have a statistically significant correlation with sociodemographic and mental health characteristics. It was assumed that career commitment and career exploration would be significantly positively correlated with school satisfaction, school success and subjective well-being and negatively correlated with general psychological distress. It was expected that career commitment and career exploration would have no correlation with gender and that they would have low positive correlations with the socioeconomic status of the family and the education of the parents.

The second goal of the second study was to more deeply examine the relationships between career exploration and career commitment, on the one hand, and between subjective well-being, general psychological distress and school satisfaction, on the other hand, via canonical correlation analysis.

The third goal was to form groups of respondents according to the status of their career identity and then determine whether they differ in sociodemographics and mental health characteristics. It was planned that, on the basis of the scores obtained by the respondents on the career identity subscales, each respondent would be assigned one of the four possible career identity statuses—CC, CB, CS or CR—in accordance with the definitions of the career identity status that were created in the theoretical part of this work. The assumption was that students with CC status would have higher levels of mental health, school satisfaction and school success and that students with CS status would have lower levels.

### 2.6. Participants and Procedures in the Second Study

The research was conducted in March 2025. At the time of the research, a total of 586 students had attended the Hospitality and Tourism School with a student dormitory in Vrnjačka Banja, Serbia. The research was conducted through the Google Forms application—a Google questionnaire—and all the research instruments were entered into this application. The researcher, the first author of this paper, was present in the classroom and informed students that they would complete a questionnaire related to their career development, emphasizing that participation was voluntary and anonymous. No further interaction between the researcher and participants occurred during questionnaire completion. Each student completed the questionnaire individually on their own device during class time. Due to the anonymous nature of the online survey, responses cannot be linked to individual participants. The questionnaire was completed by all the students who were attending classes in the school’s classrooms, i.e., they were not absent because they were attending practical classes or because of personal reasons such as illness, etc. A total of 314 students filled out the Google questionnaire.

### 2.7. Measures in the Second Study

A career identity questionnaire of vocational school students, which contains a scale of career commitment and a scale of career exploration, was used. To examine aspects of mental health, two instruments were used: the Serbian version of the short subjective well-being questionnaire (Jovanović & Novović, 2008), the original instrument constructed by Diener (2000), and the Serbian language version of DASS-42 (Depression, Anxiety and Stress Scale–42), the original DASS-42 constructed by Lovibond and Lovibond (1995).

The short scale of subjective well-being contains two subscales, positive attitude toward life and positive affectivity, each subscale has 4 items. The items of the scale—positive attitude toward life, refer to a generally positive evaluation of life and an optimistic attitude toward life, whereas the items of the positive affectivity scale refer to the experience of positive emotions in life. For each item, the respondents answered how much they agreed with the statement on a scale from 1 to 5. The total score is obtained when the answers from all the items are added and when the sum is divided by the number of items (i.e., 8). The total score is used as a measure of subjective well-being (Jovanović & Novović, 2008), which is also the case in this research.

The DASS-42 includes three subscales: depression, anxiety and stress. The depression subscale consists of items that assess the basic symptoms of depression: low positive affect, dysphoria, hopelessness, loss of interest, inertness, and negative attitudes towards oneself and life in general. The anxiety subscale includes items that primarily refer to symptoms of physiological arousal, such as dry mouth, difficulty breathing, trembling, and the subjective experience of an anxious affect. The stress subscale assesses symptoms of general, nonspecific arousal, such as difficulty relaxing and irritability. The participants estimated how often the listed symptoms occurred in their life on a scale from 0, which meant not at all, to 3, which meant mostly or almost always. The total score on the DASS-42 is used as a marker of general psychological distress (Lovibond & Lovibond, 1995), which was also the case in this research.

A special questionnaire developed for the purposes of this study was used to collect sociodemographic and school-related data. It included items on gender, age, current school grade, parents’ education, perceived family socioeconomic status, school satisfaction, and academic achievement at the end of the previous school year.

Gender was coded as 1 = male and 2 = female. Age was reported in years, and participants indicated their current grade (first to fourth year of secondary school). Parental education was assessed separately for mothers and fathers on a 9-point scale reflecting increasing levels of educational attainment, from elementary school to doctoral degree, in accordance with the educational system in the Republic of Serbia.

Perceived socioeconomic status was measured using a 5-point scale, with higher scores indicating higher levels of material resources and access to cultural, recreational, and educational opportunities.

Satisfaction with the school was examined by asking the participants to rate on a scale of 1–5 how satisfied they are with the school they are currently attending, where 1 was the lowest rating and 5 was the highest rating. When it comes to school success in the previous grade, the participants answered on the following scale: (1) unsatisfactory—I repeat the grade, (2) satisfactory, (3) good, (4) very good and (5) excellent.

### 2.8. Data Analyses in the Second Study

The following descriptive statistics were used: frequencies and percentages, minimums and maximums, means and standard deviations, skewness and kurtosis. Cronbach’s alpha coefficient was used to check the reliability of the scales. Correlation coefficients (Pearson) and their significance tests were used to describe the relationships between variables. Canonical correlation analysis was used to examine associations between sets of variables. Analysis of variance was used to determine differences between respondents with different career identities in terms of the degree of expression of mental health variables and sociodemographic characteristics. The chi-square test, Mann–Whitney U test were used for additional analyses.

In the first study, a formative model of the relationship between items and scales was assumed. The behaviors captured by the items represented formative elements of the dimensions measured by the scales. In the second study, Confirmatory Factor Analysis (CFA) was conducted. This allowed us to test a reflective model, meaning that exploration and commitment were treated as latent factors underlying item responses. In this way, we tested the validity of the model obtained in the first study in a top-down manner. A reflective model of the relationship between items and dimensions of career identity was also used in the work of Porfeli et al. (2011), who applied CFA in the validation of the VISA questionnaire. CFA was performed using Jamovi 2.6.44.

## 3. Results

### 3.1. The Results of the First Study

As mentioned, the aim of the first study was to construct a valid and reliable questionnaire of the career identity of vocational school students.

#### 3.1.1. The Sample of the First Study

The sample included students with the following educational profiles: 18 tourist technicians, 17 tourist-hotel technicians, 68 culinary technicians, 19 catering technicians, 15 hotel and restaurant technicians, 24 cooks, 13 waiters, and 14 pastry chefs; 110 female students and 78 male students participated (Table 1).

#### 3.1.2. Descriptive Item Measures

The descriptive measures for all the items are shown in Table 2. Taking into account the values of means and the low negative values of skewness, the findings indicate that the distributions of responses to the items are slightly shifted toward higher values.

#### 3.1.3. Results of Factor Analysis

First, the Kaiser–Meyer–Olkin test of sample adequacy and Bartlett’s test of sphericity showed that the items of the questionnaire are correlated with each other so that it is justified to conduct a factor analysis (KMO = 0.89; χ^2^ = 1830.44; df = 190; *p* < 0.01).

The results of the factor analysis are given in Table 3. Four factors with an eigenvalue over 1 were singled out, which together explained 61.47% of the response variance. Among those four factors, the first three are interpretable in accordance with the concept of career identity. The fourth factor includes only 5.07% of the explained variance and saturates items that already have saturation with previous factors, so it was redundant for use in the formation of scales.

In the first study, a theoretically grounded two-factor structure was initially assumed; however, analyses of the data indicated a more differentiated three-factor solution, distinguishing between exploration within and outside the educational profile alongside career commitment. Table 3 shows saturations higher than 0.3 because the rest are considered insignificant.

Items 9, 11, 19, 5, 13 and 17 are saturated exclusively with the first factor. These items are designed to represent a scale of career commitment. The first factor clearly refers to career commitment, as expected. Therefore, the listed items formed the scale of career commitment.

Items 20, 10, 6, 16 and 4 are significantly saturated with the second factor, and their content refers to the part of career exploration that concerns the current choice of educational profile, i.e., the choice of vocation. Those items mostly show the thoughtfulness of one’s vocational choice and its good alignment with one’s own self. A scale of career exploration of the chosen job vocation was created from the above items.

Items 14, 18 and 12 are saturated exclusively with the third factor. Those items refer to the exploration of career opportunities outside the existing vocational choice, i.e., outside the existing repertoire of jobs that the chosen educational profile implies. A scale of career exploration outside the selected educational profile was created from the above items.

The results are mostly expected. The findings indicate that career commitment is a homogenous construct that implies occupational preference within the educational profile. In contrast to career commitment, the results indicate that there are two processes of career exploration in students, one within the existing career direction, and the other outside the framework of the educational profile that the student attends. However, if the constructs measured by the mentioned exploration scales are correlated with a common scale greater than 0.7, those scales can be considered subscales of the common scale because the constructs measured by those scales have more than 50% of the common variance. Additionally, the mutual correlation between the career commitment scale and the exploration scale should be less than 0.7, because these constructs should differ at least to that extent.

#### 3.1.4. Correlations Between the Constructs Measured by the Scales

On the basis of the results of the factor analysis, three scales were first formed: the career commitment scale (consisting of items: 9, 11, 19, 5, 13 and 17), the exploration scale inside the chosen educational profile (items: 20, 10, 6, 16, and 4), the exploration scale outside the chosen educational profile (items: 14, 18, and 12). A career exploration scale was subsequently developed, which consists of items from both exploration scales (items: 20, 10, 6, 16, 4, 14, 18, and 12).

The correlation between exploration inside the educational profile and exploration outside the educational profile with the common scale of career exploration is greater than 0.7 (Table 4). The correlation between career commitment and the other examined phenomena was expected. Career commitment was closely related to career exploration inside the chosen educational profile, with 31.36% of the common variance (r = 0.56, *p* < 0.01), and less closely related to the exploration of occupations outside the educational profile, with 3.24% of the common variance (r = 0.18, *p* < 0.01).

The common variance in the exploration of occupations outside the educational profile and occupations inside the educational profile is 18.49% (r = 0.43, *p* < 0.01). The common variance of exploration inside the chosen educational profile and career exploration was 82.81% (r = 0.91, *p* < 0.01), whereas the common variance of exploration outside the educational profile and career exploration was 57.76% (r = 0.76, *p* < 0.01). The above results indicate that career exploration inside the chosen profile and career exploration outside the chosen educational profile are two aspects of career exploration, so one scale of career exploration is justified.

#### 3.1.5. Internal Consistency of the Scales

The internal consistency of the obtained scales was tested to check their reliability. The career commitment scale, which consists of the abovementioned 6 items, has very good reliability—Cronbach’s alpha is 0.88. The career exploration scale, which consists of the abovementioned 8 items related to career exploration inside and outside the chosen educational profile, has satisfactory reliability—Cronbach’s alpha is 0.78. The career exploration subscale, referred to as exploration inside the chosen educational profile, has satisfactory reliability, with a Cronbach’s alpha of 0.75, whereas the career exploration subscale, referred to as exploration outside the chosen educational profile, has a reliability of 0.68 (Cronbach’s alpha).

### 3.2. The Results of the Second Study

In the second study, the vocational school students’ career identity questionnaire was used in its final form, which was obtained in the first study. It included the aforementioned 14 items, 6 items for career commitment, and 8 items for career exploration. The goal was to investigate its convergent validity.

#### 3.2.1. Participant Characteristics in the Second Study

A total of 314 students were examined. The dataset contained 21 multivariate outliers, which were identified via the Mahalanobis distance due to a likelihood of occurrence of *p* < 0.001. After removing the outliers, the final sample included 293 students aged 15–19 years. The average age of the participants was 16.55 years (SD = 1.10). The sample included the following: (1) 76 first-grade students, 28 male and 48 female; (2) 85 respondents attending second grade, 33 male and 52 female; (3) 70 third-grade students, 37 male and 33 female; and (4) 62 fourth-grade students, 27 male and 35 female. Overall, there were a total of 125 male and 168 female participants.

#### 3.2.2. Descriptive Statistics of the Second Study

The descriptive statistics of the second study are presented in Table 5. The absolute values of skewness are less than 2, and the absolute values of kurtosis are less than 7, which indicates that there is no substantive nonnormality of distributions, according to Hair et al. (2022).

In the second study, the career commitment scale had an alpha coefficient value of 0.84 (Cronbach’s alpha), the career exploration scale had an alpha coefficient value of 0.71, the subjective well-being scale had a value of 0.92, and the general psychological distress scale (total score on the DASS-42) had an alpha value of 0.87. Those results show that the mentioned scales are reliable.

#### 3.2.3. Correlations Between Variables in the Second Study

The correlations between the observed variables are shown in Table 6. As expected, career exploration and career commitment were positively correlated (r = 0.36, *p* < 0.01).

Career exploration was positively correlated with school satisfaction (r = 0.31, *p* < 0.01), subjective well-being (r = 0.26, *p* < 0.01) and school success (r = 0.14, *p* < 0.05), as assumed. Career commitment was significantly positively correlated with school satisfaction (r = 0.56, *p* < 0.01), subjective well-being (r = 0.27, *p* < 0.01) and school success (r = 0.15, *p* < 0.01), which was also expected. Career commitment was negatively correlated with gender (r = −0.14; *p* < 0.05) and grade (r = −0.13; *p* < 0.05).

#### 3.2.4. Canonical Correlation Between Variables of Career Identity and Variables of Mental Health

With respect to the theory of this work, in the procedure of canonical correlation analysis, the first set of variables consisted of career commitment and career exploration because these are the processes of career identity formation. The second set of variables consisted of the following variables: satisfaction with school, subjective well-being and general psychological distress, which are variables related to mental health. The results of the canonical correlation analysis show that the first canonical correlation function is statistically significant; the canonical correlation coefficient is 0.60, the eigenvalue is 0.57, and Wilk’s Λ is 0.62 (F = 25.5; Num D.F. = 6.00; Denom D.F. = 576.00; *p* < 0.00). The second canonical correlation function is not statistically significant; its eigenvalue is 0.01. Therefore, the first function includes 97.12% of the variance of the variables entered into the analysis procedure. The data on the first canonical function are shown in Table 7.

According to the results shown in Table 7, career commitment and career exploration jointly account for 64% of the variance in the first synthetic variable (R^2^_1s_ = 0.64), which is named career identity. The findings indicate that with a decrease in the degree of career commitment or a decrease in the degree of career exploration, career identity weakens, and with a greater degree of career commitment or greater degree of career exploration, the degree of career identity is also greater. The first synthetic variable explains approximately 14% of the second synthetic variable (R^2^_21_ = 0.14), which is related to mental health. This finding indicates that with a lower level of career identity development, the level of mental health is also lower. Additionally, with a greater degree of career identity, mental health is better. School satisfaction, subjective well-being and general distress accounted for 40% of the variance in the second synthetic variable (R^2^_2s_ = 0.40). However, general psychological distress has negligible saturation with the second synthetic variable (r_s_ = 0.14). Therefore, in this constellation of variables, the synthetic variable related to mental health is determined by subjective well-being and satisfaction with school so that a higher degree of satisfaction with school and a higher degree of subjective well-being imply a greater degree of mental health. Additionally, with declining satisfaction with school or declining subjective well-being, mental health also weakens. On the other hand, 23% of the variance of career identity is explained by the synthetic variable related to mental health (R^2^_12_ = 0.23). This finding indicates that good mental health is an important condition for a well-developed career identity.

Cross loadings show how each variable of one set is correlated with another synthetic variable. The findings indicate that career commitment (r_cs_ = −0.36) significantly explains the variance in the second synthetic variable, mental health, in the same direction as career exploration does (r_cs_ = −0.58). With a lower degree of career commitment or with a lower degree of career exploration, mental health also declines. Satisfaction with school (r_cs_ = −0.57) and subjective well-being (r_cs_ = −0.31) significantly explain the variance of the synthetic variable—career identity. With a lower level of satisfaction with school or with a lower level of subjective well-being, career identity weakens.

#### 3.2.5. Differences Between Students with Different Career Identity Statuses

**Figure 1:**
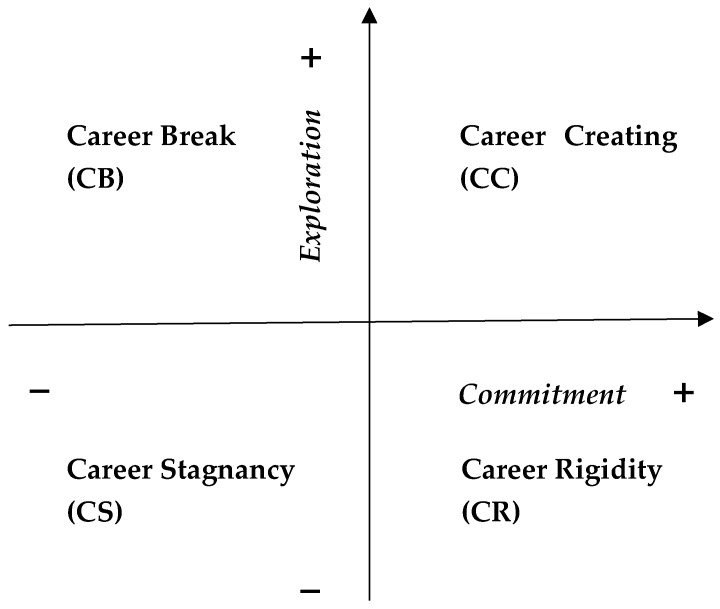
Graphical display of career identity statuses.

Given that career commitment and career exploration represent dimensions, each participant was assigned one of the four career identity statuses by crossing those dimensions. To do this in the easiest way, first the participants’ raw scores were transformed into z scores. Students were assigned status CC (Career Creating) if their z scores on both scales of the Career Identity Questionnaire were greater than zero. Students are assigned Status CB (Career Break) if their z score on the career commitment scale is less than or equal to zero and if their z score on the career exploration scale is greater than zero. The status CR (Career Rigidity) was indicated by a z score on the career commitment scale greater than 0 and a z score on the career exploration scale less than or equal to zero. The status CS (Career Stagnation) meant that the z scores on both scales were less than or equal to zero (Figure 1).

The results related to the classification of students according to career identity status are as follows: (1) a total of 111 students had CC status (37.90%); (2) CB status was held by 48 students (16.40%); (3) CS status was held by 87 students (29.70%); and (4) CR status was held by 47 students (16.00%). There were 55 male and 56 female students with CC status, 14 male and 34 female students with CB status, 32 male and 55 female students with CS status, and 24 male and 23 female students with CR status. The chi-square test (χ^2^) was used to check the statistical significance of the differences in the frequencies of the respondents’ gender in certain career identity statuses. With respect to CB status there were significantly more girls than boys (χ^2^ = 8.33; *p* < 0.01); with respect to CS status there were significantly more girls than boys (χ^2^ = 6.08; *p* < 0.05), and no significant differences were found in the remaining two statuses.

To analyze the differences in the degree of expression of other variables for students with different career identity statuses, Levene’s homogeneity of variance test was first performed. For the variable general psychological distress, Levene’s test revealed that the variance was not homogeneous across the groups of respondents according to their career identity status (Levene statistic = 4.84; df1 = 3, df2 = 289; *p* < 0.05). Therefore, the Mann–Whitney U test was used to examine the differences in the degree of general distress between students with different career statuses. Each status was compared with each other, there were 6 combinations and 6 tests were performed. The mean values of general psychological distress for groups of students with different career identity statuses were as follows: for CC status, 45.83 (SD = 26.23); for CB status, 47.89 (SD = 27.53); for CS status, 55.65 (SD = 31.47); and for CR status, 42.02 (SD = 21.55). The following was found: students with CC status had significantly less general psychological distress than did students with CS status (U = 5681.00; *p* < 0.05); students with CR status had significantly less general psychological distress than did students with CS status (U = 1552.20; *p* < 0.05); other differences were not statistically significant.

An F test of analysis of variance was performed to examine differences in other variables. Statistically significant differences were revealed between students with different career identity statuses, as shown in Table 8.

Only when it comes to father’s education, students with different career status did not statistically significantly differ (F = 0.62; *p* > 0.60). For subsequent tests (post hoc tests), Tukey’s HSD test was used. Tukey’s test revealed that students with CB status were significantly older than students with CS status (*p* < 0.05); students with CB status were also significantly older than students with CR status (*p* < 0.05). Students with CB status were attending higher grades than students with CC status (*p* < 0.05); students with CB status were attending higher grades than students with CS status (*p* < 0.05), too.

With respect to the mother’s education and the socioeconomic status of the family, Tukey’s test revealed that respondents with CB status reported an average higher level of mother’s education than respondents with CS status (*p* < 0.05), and respondents with CC status reported a more favorable socioeconomic status of the family than respondents with CR status (*p* < 0.05).

Tukey’s tests also revealed that students with CC status had better academic performance than students with CS status (*p* < 0.05); students with CC status were more satisfied with school than students with CS (*p* < 0.05), and students with CC status were more satisfied with school than students with CB (*p* < 0.05). Students with CR status reported greater satisfaction with school than students with CS status (*p* < 0.05), and students with CR status reported greater satisfaction with school than students with CB status (*p* < 0.05).

With respect to subjective well-being, students with CC status had significantly more favorable subjective well-being compared to students with CS status (*p* < 0.05) and CR status (*p* < 0.05). Other differences were not significant.

#### 3.2.6. Results of the Confirmatory Factor Analysis of the Career Identity Questionnaire in the Second Study

Given that in the first study we obtained three main components that create the items of the vocational school students’ career identity questionnaire, we tested that three-factor solution through CFA. As a reminder, the first factor related to exploration within the chosen educational profile (items 1, 3, 5, 11, and 14), the second factor concerned exploration outside the chosen educational profile (items 7, 9, and 13), and the third factor pertained to career commitment (items 2, 4, 6, 8, 10, and 12).

The results of the CFA indicate that the initial three-factor model did not achieve an adequate level of fit to the data (χ^2^(74) = 232, *p* < 0.001; CFI = 0.87; TLI = 0.84; RMSEA = 0.08; SRMR = 0.06). Although the SRMR value suggests acceptable fit, the remaining indices (particularly CFI and TLI, which are below the recommended 0.90 threshold) indicate that the model requires further improvement (Table 9).

Following model modifications, which involved allowing covariances between the residuals of items 5 and 9, 5 and 14, 8 and 2, and 8 and 6, a substantially better fit was obtained (χ^2^(69) = 145, *p* < 0.001; CFI = 0.93; TLI = 0.91; RMSEA = 0.06; SRMR = 0.05). All key fit indices in the modified model reach or exceed recommended cutoff values, with RMSEA and SRMR indicating good fit, and CFI and TLI suggesting acceptable to very good model fit.

These findings suggest that the three-factor structure of the questionnaire—comprising commitment, exploration within the educational profile, and exploration outside the educational profile—represents an adequate model for the data. It should be noted that achieving optimal fit required accounting for certain associations between items that are not fully explained by the latent factors, which most likely reflects shared unexplained variance due to linguistic similarity among the items.

Standardized factor loadings (λ) are generally moderate to high, ranging approximately from 0.40 to 0.78, indicating that most items adequately load on their respective latent factors (Table 10). Comparison of the initial (λ) and modified (λ′) models indicates stable factor loadings, with most indicators showing slight improvements following model modifications. The highest loadings were observed for items measuring career commitment, where factor loadings are consistently high (approximately 0.65 to 0.75), suggesting a stable and homogeneous latent construct. Corrected item–total correlation values are generally acceptable, ranging from approximately 0.30 to 0.67, indicating that most items contribute meaningfully to the explanation of the overall construct, although certain items within the exploration factors demonstrate weaker discriminative power relative to the total scale. Composite reliability (CR) shows that the career commitment factor achieved a value of 0.85, indicating high internal consistency. The exploration within the educational profile factor reached a composite reliability of 0.69, while the exploration outside the educational profile factor showed a value of 0.60, suggesting acceptable but comparatively lower reliability for both dimensions. Average variance extracted (AVE) indicates that the career commitment factor reached a value of 0.49, which is close to the recommended threshold of 0.50. In contrast, the exploration within the educational profile factor showed a value of 0.32, while the exploration outside the educational profile factor reached 0.35, suggesting that both exploration dimensions explain a relatively smaller proportion of variance in their indicators.

Mean inter-item correlation (MIIC) values further support the observed differences in psychometric quality across the three dimensions. The exploration within the educational profile scale showed a MIIC of 0.30, while the exploration outside the educational profile scale reached 0.31, both indicating acceptable levels of internal consistency. The career commitment scale demonstrated a higher MIIC of 0.48, suggesting stronger interrelatedness and greater homogeneity among its items. These findings are consistent with previous results based on CR and AVE, confirming that the career commitment construct is more internally coherent, whereas the exploration dimensions capture broader and more heterogeneous aspects of the construct.

## 4. Discussion

### 4.1. Discussion of the First Study

The results of the principal component analysis provided partial support for the hypothesized two-factor structure, with career commitment emerging as a distinct factor and career exploration differentiating into two related dimensions (within and outside the educational profile). This suggests that career exploration may represent a more nuanced construct than initially assumed, while still forming a coherent domain. Based on the results of the first study, a career identity questionnaire was developed comprising two scales: career exploration and career commitment. As expected, the final version included a reduced number of items compared to the initial pool, while both scales demonstrated satisfactory reliability (α = 0.88 and α = 0.78). The two constructs were moderately and positively correlated (r = 0.48, *p* < 0.01), which is consistent with theoretical perspectives that conceptualize exploration and commitment as interrelated processes of identity development (Meeus et al., 2002). This finding is also in line with career development theories emphasizing adolescence as a period of active exploration of career opportunities (Amundson et al., 2015; Super, 1980; Super et al., 1996). Although exploration was operationalized as a single scale in the final instrument, its internal structure reflects two closely related domains—within and outside the educational profile—indicating that students’ exploratory behavior encompasses both engagement within their current vocational pathway and consideration of alternative career options. A significant positive correlation between career commitment and exploration outside the educational profile (r = 0.18, *p* < 0.01) indicates that students who are committed to their chosen career path also explore other career paths to some extent. The exploration of a career outside the chosen educational profile is reminiscent of an earlier concept called reconsideration of commitment (Meeus et al., 2012; Crocetti, 2017), which implies considering the compatibility of other career paths with one’s own personality, comparing them with the current path, and recognizing the advantages of those new career paths over the current paths. However, in relation to the reconsideration of commitment, the exploration of a career path outside the chosen educational profile is more related to the creation of “plan B” as a backup option for further education or entering the world of work without diminishing the importance of the current career commitment.

Exploration of the career inside the chosen educational profile somewhat overlaps with the term in-depth career exploration (Meeus et al., 2012; Crocetti, 2017), which implies insight into why one should stay on the chosen career path. However, in contrast to that term, career exploration within the chosen educational profile refers to getting to know possible education or work options within the existing career path.

Both career exploration subscales are more strongly correlated with the career exploration scale than 0.7 is, so they can be treated as two aspects of the same phenomenon. The career exploration subscales can be used to understand the structure of results in individual work with adolescents because these subscales provide insight into the extent to which the adolescent explores career paths within or outside the framework of his or her chosen educational profile.

The results of this study, as well as the theories and practices of career guidance and counseling, emphasize that career exploration and career commitment are complex and interdependent processes (Amundson et al., 2015; Meijers, 1998; Super et al., 1996; Savickas, 2002, 2005; Krumboltz, 1996). Career commitment depends on acquired knowledge, skills and positive attitudes related to the chosen vocation, for which quality exploration of both oneself and the environment is necessary. This exploration is usually successful when there is a prior commitment to schooling or work.

The items of the career commitment scale are consistent with Erikson’s idea that commitment requires the virtue of loyalty, which is based on all previously acquired ego virtues (Erikson, 1968). Career commitment includes items related to the hope that in the future the acquired knowledge from professional subjects and from practical classes will be useful (“The knowledge I acquire from vocational subjects will help me a lot in my future job”); then, they refer to the experience of one’s own diligence and competence in practical classes or training (“The teachers of vocational subjects have confidence in me that I will complete the tasks they set for me successfully and on time”), which also include items that indicate the experience of purpose and initiative in practical classes or training (“I can see myself with satisfaction doing the job I’m currently training for after completing my education”, “Other people can see my positive energy while I’m gaining professional skills in practical classes”).

### 4.2. Conclusions of the First Study

Using factor analysis, the original number of 20 items that made up the career identity questionnaire of vocational school students was reduced to 14. A career identity questionnaire was created that has factorially valid and reliable scales intended to measure career commitment and career exploration, which are presented as concepts in the introductory part of this paper.

### 4.3. Discussion of the Second Study

In the second study, the vocational school students’ career identity questionnaire was used in its final form, which was obtained in the first study. The results of the correlation analysis revealed that career exploration was significantly correlated with career commitment (r = 0.36, *p* < 0.01), satisfaction with school (r = 0.31, *p* < 0.01), subjective well-being (r = 0.26, *p* < 0.01) and school success (r = 0.14, *p* < 0.05). These findings indicate that adolescents with greater career exploration have greater career commitment, greater satisfaction with school, higher levels of subjective well-being, and better school success. Career commitment was significantly correlated with satisfaction with school (r = 0.56, *p* < 0.01), subjective well-being (r = 0.27, *p* < 0.01), school success (r = 0.15, *p* < 0.01), gender (r = −0.14, *p* < 0.05) and grade (r = −0.13, *p* < 0.05). These findings indicate that career commitment increases with school satisfaction, subjective well-being, and academic achievement and that career commitment decreases slightly as participants move into higher grades of school and slightly decreases with female gender. Career exploration does not contribute to a reduction in existing commitment due to the search for a different career identity. In contrast, career exploration and career commitment are parallel processes that support each other. As already mentioned, career guidance and counseling theories describe adolescence as the developmental stage in which career possibilities are explored the most (Amundson et al., 2015; Super, 1980; Super et al., 1996). Because career commitment includes learning motivation and school engagement, some skills needed for career exploration are acquired through career commitment. This is also an important reason for the significant positive correlation between career commitment and career exploration.

The Meeus–Crocetti model (Crocetti et al., 2008; Crocetti & Meeus, 2014) conceptualizes identity formation through three interrelated processes: commitment, in-depth exploration, and reconsideration of commitment, whose interaction yields five identity statuses (achievement, foreclosure, moratorium, searching moratorium, and diffusion). This framework assumes that identity development unfolds dynamically over time through fluctuations and transitions among statuses. In contrast, the present study conceptualizes career identity development in vocational education as a context-bound and organized process. It assumes that adolescents are already engaged in career construction within structured educational pathways and that the primary developmental task is the organization of career identity in response to increasing awareness of multiple career options. Accordingly, four functional career identity statuses (CC, CR, CB, CS) are identified, reflecting distinct configurations of career commitment and exploration within and outside the chosen educational profile. Consequently, future research should examine the development of these career identity statuses, which would require further refinement and validation of the measurement instrument.

The findings of the present study are broadly consistent with the multidimensional conceptualization of vocational identity status proposed by Porfeli et al. (2011), who emphasize the dynamic interplay between commitment and exploration processes. Similarly, the present results indicate that career commitment and exploration represent interconnected but distinct dimensions of career identity. However, unlike the Porfeli model, which generally conceptualizes exploration in terms of breadth, depth, commitment (making and identification), and reconsideration (self-doubt and flexibility), the current study differentiates commitment and exploration within and outside the chosen educational profile, reflecting the specific context of vocational education. The present study is in line with previous works (Porfeli et al., 2011; Šimunović et al., 2026) by demonstrating that career identity is significantly associated with indicators of mental health, thereby highlighting the functional relevance of career identity in adolescent development.

All the virtues of the ego that Erikson wrote about enable exploration and commitment (Erikson, 1968). According to the theory of Erikson, the successful resolution of an individual’s identity crisis depends on his or her basic ego virtues, which start from the earliest developmental virtue to the virtue that emerges in the period of adolescence: the virtue of hope (trust), the virtue of autonomy (will), the virtue purpose (initiative), the virtue of diligence (competence) and loyalty. In his time, Erikson assumed a conflict between the demands of the social environment about what kind of individual it wanted, on the one hand, and the individual’s desire, on the other hand. Today, in the modern culture of the Western world, it is difficult to address this kind of conflict because the freedom of choice of the individuals to develop themselves according to their abilities and interests is emphasized. At this time, the adolescent crisis does not refer to the conflict between the social environment and the individual, as Erikson wrote. The youth crisis today involves finding the options that best suit the individual. Both exploration and commitment are necessary processes, at the same time. The finding of this study concerning the positive correlation between career exploration and career commitment is in accordance with the idea that students who possess the virtues of hope, will, initiative and industry, have loyalty directed towards the chosen career path, which is the basis of their career commitment. Additionally, their virtue of loyalty manifests toward themselves as freely self-actualizing beings by choosing career paths that suit them and using career exploration. Career commitment and exploration grow together and represent the manifestation of the mentioned virtues of the individual in the field of education and work.

All the abovementioned modern theories of career guidance and counseling indicate that the client should show diligence and loyalty in joint work with the career counselor (Super et al., 1996; Savickas, 2002, 2005; Holland, 1997; Krumboltz, 1996). The client’s virtues of hope, will, purpose and industry play a significant role in every topic of the client’s work with a career counselor related to finding and building a career path that suits the client.

The finding that career commitment slightly decreases with higher grades indicates the specifics of the sample of this research: some students, as they grow up and move to higher grades, achieve better knowledge of themselves and the chosen educational profile, so that their commitment to the career path related to the chosen educational profile decreases. The finding that there is a slight drop in career commitment among the girls in this sample indicates that girls reconsider their choice of educational profile more than boys do and thus find more flaws in it.

As expected, the results of the canonical correlation analysis revealed that career commitment and career exploration form a variable called career identity. Subjective well-being and satisfaction with school are related aspects of mental health and are related to interdependence with career identity. Earlier works indicate that there is a significant positive correlation between subjective well-being and satisfaction with school, which is an aspect of school engagement (Shu et al., 2024; Keijzer et al., 2022), whereas other previous works indicate that subjective well-being is significantly positively correlated with successful career development (Brown & Lent, 2021; Amundson et al., 2015; Krumboltz, 1996; De Lise et al., 2024; Redekopp & Huston, 2018; Karas et al., 2015; Skorikov & Vondracek, 2011; De Goede et al., 1999).

According to the results of the canonical correlation analysis, general psychological distress, which includes anxiety, depression and negative stress, was not a variable with a significant correlation with career identity. This finding is not in line with previous works indicating that difficulties in career development can be related to anxiety, depression and stress (Krumboltz, 1996; Redekopp & Huston, 2018; Amaral et al., 2023; Dik et al., 2022; Hardin et al., 2006; Kulcsár et al., 2024). These papers emphasize that when individuals have serious difficulties in creating their own career path, they usually also have serious difficulties with their mental health. However, the students included in this research, considering that they were attending vocational school, had already chosen a career path related to tourism and hospitality, which means that they were not truly without any career choice.

It was hypothesized that the most desirable form of career identity is CC status. Participants with scores above average on both scales of the Career Identity Questionnaire were given this status. Most of them are in the sample (37.90% of the sample), as was assumed, because career–creating status is the assumed norm of healthy development, as stated in the introduction of this paper. Compared with students who have CS status, those with CC status have greater subjective well-being, better achievement in school and greater satisfaction with school. This finding is in line with previous research indicating that the good mental health of adolescents also includes the successful development of a career identity (Amundson et al., 2015; Krumboltz, 1993, 1996; De Lise et al., 2024; Redekopp & Huston, 2018; Karas et al., 2015; Skorikov & Vondracek, 2011; De Goede et al., 1999), as well as greater engagement in school (Shu et al., 2024; Keijzer et al., 2022). Additionally, students with CC status reported a more favorable socioeconomic status of their families than students with CS status.

Students with CR status (16%) have greater satisfaction with school than students with CS status, but they do not have as high a degree of subjective well-being as do students with CC status. Rigidity in accepting a career path in students with CR status implies a developed career commitment without developed career exploration, which implies a weaker experience of subjective well-being than in students with CC status, but still less general psychological distress than in students with CS status. No statistically significant differences were found between students with CR status and students with CB status in terms of mental health characteristics. The development of career commitment, without the development of career exploration, indicates the possible imposition of a career path by important persons, which reminiscent of the concept of foreclosure identity from earlier works (Erikson, 1968; Marcia, 1980). However, foreclosure identity and CR status are different concepts and their relationships should be examined in other studies.

Students with CB status (16.40%), i.e., students with below-average commitment and above-average exploration, are significantly less satisfied with school than are students with CC and CR status. They make greater efforts to explore new career opportunities, so they are also less committed than average. It is as if they are taking a break in their commitment, hence the name of this status—a career break. The indicators of their mental health vary so much that in this respect there are no significant differences compared with other groups of students that were formed according to the status of their career identity. However, some differences were discovered. The students with CB status in this sample are older than the students with CC and CS statuses and are also in higher grades than students with CR and CS statuses. This indicates that during their education, i.e., as they move from grade to grade, they discovered that their current career path was not good enough for them, so they are now looking for a new path. In this sample, there were significantly more girls than boys among adolescents with CB status, and their mothers’ education was significantly greater than that among students with CS status. CB status resembles the concept of moratorium identity (Erikson, 1968; Marcia, 2007), where there is low commitment and high exploration. However, CB status and moratorium identity are different concepts, and their connection should be examined in other research.

The largest number of statistically significant differences was found when the group of students who experienced career stagnation was compared with other groups of students. Students with CS status have significantly more pronounced general psychological distress than students with CC and CR status. Students with CS status constitute 29.70% of the sample, as stated. The findings showed that students with CS status experienced difficulties in career development and difficulties related to anxiety, depression and negative stress, which is consistent with previous research (Amaral et al., 2023; Dik et al., 2022; Hardin et al., 2006; Kulcsár et al., 2024). Compared with students with CC status, those with CS status have weaker indicators of mental health and school success. CS status resembles the concept of identity diffusion (Erikson, 1968; Marcia, 2007), where both commitment and exploration are weak. However, the concepts of CS status and identity diffusion are different and their relationships should be examined in other studies.

The CFA results and item-level analysis provide partial support for the proposed measurement model. The three-factor structure is theoretically consistent, and most items show acceptable loadings and item–total correlations. The career commitment dimension demonstrates the strongest psychometric performance, reflected in higher loadings and satisfactory composite reliability. Consistent with this pattern, inter-item associations suggest greater homogeneity of the career commitment scale compared to the two exploration dimensions. Composite reliability values for the exploration scales are lower but still within acceptable limits, indicating moderate internal consistency relative to the more robust career commitment construct. The exploration dimensions exhibit weaker psychometric properties overall, including lower composite reliability and average variance extracted. AVE values below 0.50 suggest limited convergent validity; however, this may reflect the conceptual breadth of exploration constructs, which can reduce shared variance among indicators, particularly in early stages of scale development (Hair et al., 2022). Overall, the instrument demonstrates satisfactory structural validity, with career commitment as its main strength, while the exploration scales require further refinement through improved item specification and validation on independent samples.

### 4.4. Conclusions of the Second Study

The findings provide evidence supporting the validity and reliability of the questionnaire. Career exploration and career commitment are interconnected processes that form the career identity of vocational school students. Career exploration and career commitment of students are significantly related to their mental health. Good mental health encourages career exploration and career commitment, while at the same time the creation of career identity improves mental health. The results of the second study confirmed that the scales of the career identity questionnaire of vocational school students are valid instruments for measuring career exploration and career commitment. Students’ school satisfaction is significantly related to their subjective well-being, and these two variables together form one part of their mental health that is significantly positively correlated with their career identity.

School curricula related to career guidance and counseling should encourage career exploration and career commitment among students. The school curriculum should, among other things, include procedures related to the development of virtues in students that are discussed in Erikson’s theory (Erikson, 1968). Given that career exploration includes career exploration within and outside the framework of the chosen educational profile, it is desirable that the school’s career guidance and counseling program in vocational school enables students to have at least an alternative to their main career path.

### 4.5. Limitations of These Studies and Directions for Further Research

A potential limitation of the study is the presence of the researcher in the classroom during data collection, which may have introduced a degree of social desirability bias or an authority effect. However, all data were collected using a fully anonymous, self-administered online questionnaire completed individually on students’ own devices, and responses could not be linked to individual participants. This procedural design reduces, but does not eliminate, the potential influence of the researcher’s presence.

Although the standardized factor loadings and corrected item–total correlations generally indicate an acceptable level of item performance, the overall pattern of results suggests unequal psychometric quality across the three latent factors. While the career commitment dimension demonstrates satisfactory composite reliability and relatively stronger convergent validity, the two exploration dimensions show weaker psychometric properties, reflected in lower composite reliability values and insufficient average variance extracted. These findings indicate that the explorations within and outside the educational profile factors are measured with a lower degree of precision and internal coherence compared to the career commitment construct. In addition, the relatively low average variance extracted values suggest that a substantial proportion of variance in the indicators remains unexplained by their respective latent factors, which raises concerns regarding the convergent validity of these dimensions. Accordingly, the observed results point to a need for further refinement of the exploration scales. Future improvements should focus on revising or eliminating items with weaker psychometric performance, enhancing the conceptual clarity and specificity of the exploration construct, and potentially expanding the item pool to achieve better content coverage. Further validation on independent samples is also recommended in order to examine the stability of the factorial structure and strengthen the generalizability of the instrument.

The generalizability of all findings of this study, including those concerning the relationship between career identity and aspects of mental health, is limited, as the sample was drawn from a single vocational school. This represents an important limitation; therefore, future research should examine the robustness of these findings across multiple vocational schools and diverse educational contexts. At the same time, the Career Identity Questionnaire for Vocational School Students was designed with sufficiently generalizable items, allowing its application across different types of vocational schools. The concepts of career identity presented in this study are applicable to vocational students broadly. Future research may also examine connections with more general identity constructs, such as identity diffusion, moratorium, foreclosure, and achievement. In addition, the relationship between career identity and school dropout could be explored, given that a well-developed career identity may motivate successful educational outcomes.

This work is the first to define and measure career identity specifically for vocational school students. The developed questionnaire provides a reliable and practical tool for researchers and educators to assess and support career development, with potential benefits for students’ academic engagement and mental health. By offering a validated instrument tailored to vocational students, this study contributes a foundational resource for both research and applied practice, highlighting the significance of career identity in adolescent development and well-being.

## Figures and Tables

**Table 1:** The sample of the first study—age and gender.

		Age					Total
		15	16	17	18	19	
Gender	Female	29 (15.43%)	23 (12.23%)	32 (17.02%)	22 (11.70%)	4 (2.13%)	110 (58.51%)
	Male	14 (7.45%)	25 (13.30%)	22 (11.70%)	14 (7.45%)	3 (1.60%)	78 (41.49%)
Total		43 (22.88%)	48 (25.53%)	54 (28.72%)	36 (19.15%)	7 (3.73%)	188 (100%)

**Table 2:** Descriptive statistics of items.

Item	Mean	SD	Skew.	Kurt.
1. I feel satisfaction to attend my current school.	3.63	1.37	−0.69	−0.68
2. When I make a decision about my further education, I first independently inform myself about everything I am interested in.	4.20	1.10	−1.32	1.00
3. The teachers of vocational subjects have confidence in me that I will complete the tasks they set for me successfully and on time.	3.92	1.31	−1.01	−0.14
4. It’s interesting to me to find information about various vocations on the Internet.	3.63	1.35	−0.64	−0.73
5. I feel more satisfaction when I study material from vocational subjects than from other subjects.	3.76	1.41	−0.80	−0.71
6. Before entering high school, I tried to get as much information as possible about different schools that interested me.	3.50	1.47	−0.47	−1.13
7. I possess a lot of gift for the profession that I am now schooling for.	3.84	1.23	−0.90	−0.16
8. I consider that I will change several vocations in my future.	3.20	1.49	−0.14	−1.37
9. I can see myself with satisfaction doing the job I’m currently training for after completing my education.	3.66	1.52	−0.72	−0.99
10. I considered the pros and cons of my current school before enrolling.	3.35	1.47	−0.34	−1.26
11. The knowledge I acquire from vocational subjects will help me a lot in my future job.	3.90	1.35	−0.95	−0.38
12. I have a gift for some vocations beside the one I am currently schooling for.	3.87	1.28	−0.86	−0.36
13. I feel very proud when I acquire knowledge in vocational subjects.	3.91	1.33	−1.04	−0.09
14. I’m researching whether jobs outside of the profession I’m currently training for are suitable for me.	3.53	1.40	−0.48	−1.03
15. My current education is the best path towards the future I am planning to have.	3.52	1.42	−0.54	−1.01
16. I am informing myself about the different possible directions of my future career.	3.99	1.22	−0.90	−0.32
17. Other people can see my positive energy as I gain professional skills in practical classes.	3.86	1.37	−0.92	−0.43
18. I am interested in the possibilities of further education outside the profession which I am currently studying for.	3.40	1.51	−0.40	−1.28
19. The teachers see that I am the right person for the profession I am studying for.	3.53	1.33	−0.50	−0.84
20. Before I enrolled in high school. I discussed with other people how capable I was of successfully attending it.	3.53	1.50	−0.58	−1.10

**Table 3:** Factor analysis of initial questionnaire.

Items	Factor			
	1	2	3	4
9. I can see myself with satisfaction doing the job I’m currently training for after completing my education.	0.77			
11. The knowledge I acquire from vocational subjects will help me a lot in my future job.	0.76			
19. The teachers see that I am the right person for the profession I am studying for.	0.76			
5. I feel more satisfaction when I study material from vocational subjects than from other subjects.	0.75			
15. My current education is the best path towards the future I am planning to have.	0.74	0.36		
3. The teachers of vocational subjects have confidence in me that I will complete the tasks they set for me successfully and on time.	0.71			0.33
13. I feel very proud when I acquire knowledge in vocational subjects.	0.70			
17. Other people can see my positive energy as I gain professional skills in practical classes.	0.69			
7. I possess a lot of gift for the profession that I am now schooling for.	0.65	0.30		
1. I feel satisfaction to attend my current school.	0.58			0.40
20. Before I enrolled in high school. I discussed with other people how capable I was of successfully attending it.		0.76		
10. I considered the pros and cons of my current school before enrolling.		0.69		
6. Before entering high school. I tried to get as much information as possible about different schools that interested me.		0.56		
16. I am informing myself about the different possible directions of my future career.		0.53		0.39
14. I’m researching whether jobs outside of the profession I’m currently training for are suitable for me.			0.74	
18. I am interested in the possibilities of further education outside the profession which I am currently studying for.			0.70	
8. I consider that I will change several vocations in my future.	−0.32		0.69	
12. I have a gift for some vocations beside the one I am currently schooling for.			0.60	
2. When I make a decision about my further education. I first independently inform myself about everything I am interested in.				0.88
4. It’s interesting to me to find information about various vocations on the Internet.		0.31		0.63
eigenvalue	7.46	2.58	1.22	1.01
% explained variance	37.34	12.92	6.14	5.07

**Table 4:** Correlations between career identity constructs.

	Career Commitment	Career Exploration Inside the Chosen Educational Profile	Career Exploration Outside the Chosen Educational Profile
Career exploration inside the chosen educational profile	0.56 **		
Career exploration outside the chosen educational profile	0.18 **	0.43 **	
Career exploration	0.48 **	0.91 **	0.76 **

** the correlation is significant at the 0.01 level. Statistically significant results are bolded.

**Table 5:** Descriptive statistics in the second study.

Variable	Min	Max	Mean	SD	Skew.	Kurt.
Age	15	19	16.55	1.10	0.12	−1.06
Grade	1	4	2.40	1.08	0.13	−1.27
Education of Mother	1	9	3.09	1.38	1.35	2.38
Education of Father	1	9	3.02	1.46	1.38	2.88
SES Family	1	5	3.11	1.01	−0.19	−0.87
School Success	2	5	3.83	0.74	−0.17	−0.31
School Satisfaction	1	5	3.42	1.01	−0.46	−0.13
Career Exploration	1.00	5.00	3.46	0.72	−0.16	−0.33
Career Commitment	1.00	5.00	3.59	0.93	−0.55	−0.19
Subjective Well-Being	1.00	5.00	3.81	0.93	−0.80	0.02
General Psychological Distress	6.00	126.00	48.47	27.85	0.53	−0.40

**Table 6:** Correlations between variables in the second study.

Variable	1.	2.	3.	4.	5.	6.	7.	8.	9.	10.	11.
1. Gender	1										
2. Age	−0.07	1									
3. Grade	−0.80	0.93 **	1								
4. E. Mother	−0.13 **	−0.03	−0.00	1							
5. E. Father	−0.07	−0.08	−0.08	0.47 **	1						
6. SES Family	−0.01	0.00	−0.02	0.09	0.07	1					
7. School Succ.	0.12 **	−0.08	−0.06	−0.00	0.06	0.04	1				
8. S. Satisfaction	0.00	−0.13 *	−0.16 **	−0.08	0.06	0.04	0.14 *	1			
9. C. Explor.	−0.03	0.10	0.10	0.01	0.06	0.07	0.14 *	0.31 **	1		
10. C. Commit.	−0.14 *	−0.08	−0.13 *	−0.06	0.07	−0.08	0.15 **	0.56 **	0.36 **	1	
11. Subj. W. B.	−0.15 *	0.19 **	0.17 **	0.00	0.00	0.23 **	−0.02	0.27 **	0.26 **	0.27 **	1
12. G. P. Disstres	0.23 **	0.05	0.06	−0.14 *	−0.08	−0.27 **	−0.01	−0.14 *	−0.04	−0.08	−0.44 **

* the correlation is significant at the 0.05 level, ** the correlation is significant at the 0.01 level. Statistically significant results are bolded.

**Table 7:** The canonical solution—career identity and mental health of students.

Variable	Canonical Function 1			
Set 1:	Coeff.	C. Loadings (r_s_)	r_s_^2^%	Cross Loadings (r_cs_)
**Career Exploration**	**−0.29**	**−0.60**	**36**	**−0.36**
**Career Commitment**	**−0.85**	**−0.96**	**92.16**	**−0.58**
Set 1 by Self. Proportion of Variance Explained (R^2^_1s_):				0.64
Set 2 by Set 1. Proportion of Variance Explained (R^2^_21_):				0.14
Set 2:	Coeff.	C. Loadings (r_s_)	r_s_^2^%	Cross Loadings (r_cs_)
**School Satisfaction**	**−0.88**	**−0.95**	**90.25**	**−0.57**
**Subjective Well-Being**	**−0.33**	**−0.52**	**27.04**	**−0.31**
General Psych. Distress	−0.13	0.14	1.96	0.08
Set 2 by Self. Proportion of Variance Explained (R^2^_2s_):				0.40
Set 1 by Set 2. Proportion of Variance Explained (R^2^_12_):				0.23

Note: Statistically significant results are bolded.

**Table 8:** Differences between students who have different career identity status.

	Career Identity Status—Mean ± SD				Test	
Variables	CC	CB	CS	CR	F (289,3)	*p*
**Age**	**16.55 ± 1.12**	**16.96 ± 1.05**	**16.45 ± 1.07**	**16.30 ± 1.06**	**3.34**	**0.02**
**Grade**	**2.37 ± 1.12**	**2.85 ± 1.05**	**2.36 ± 1.05**	**2.11 ± 0.98**	**4.12**	**0.00**
**Edu. Mother**	**3.00 ± 1.29**	**3.58 ± 1.59**	**2.93 ± 1.16**	**3.11 ± 1.63**	**2.61**	**0.05**
Edu. Father	3.15 ± 1.57	3.04 ± 1.32	2.87 ± 1.19	2.96 ± 1.78	0.62	0.60
**SES Family**	**3.20 ± 0.95**	**3.19 ± 1.04**	**3.16 ± 1.11**	**2.70 ± 0.97**	**3.02**	**0.03**
**S. G. Average**	**3.95 ± 0.67**	**3.83 ± 0.80**	**3.62 ± 0.82**	**3.96 ± 0.58**	**3.77**	**0.01**
**S. Satisfaction**	**3.89 ± 0.89**	**3.10 ± 0.83**	**2.84 ± 0.99**	**3.72 ± 0.80**	**25.75**	**0.00**
**Subj. W. B.**	**4.15 ± 0.77**	**3.79 ± 0.93**	**3.47 ±1.00**	**3.65 ± 0.88**	**10.02**	**0.00**

Note: Edu. Mother—Education of Mother, Edu. Father—Education of Father, SES Family—Socio—economical Status of Family, S. G. Average—School Grade Average; S. Satisfaction—School Satisfaction; Subj. W. B.—Subjective Well-Being. Statistically significant results are bolded.

**Table 9:** Fit indices for a model that includes commitment and explorations within and outside the educational profile.

Model	χ^2^	df	*p*	CFI	TLI	RMSEA [90% CI]	SRMR
Initial model	232	74	<0.00	0.87	0.84	0.08 [0.07, 0.09]	0.06
Modified * model	145	69	<0.00	0.93	0.91	0.06 [0.04, 0.07]	0.05

Note: * Covariances were allowed between residuals of items 5-9, 5-14, 8-2, and 8-6 due to semantic similarities.

**Table 10:** Standardized factor loadings and corrected item–total correlations.

Factor	Item	λ′ (λ)	CITC
Factor 1: Exploration inside education profile	1. It’s interesting to me to find information about various vocations on the Internet.	0.42 (0.42)	0.35
	3. Before entering high school, I tried to get as much information as possible about different schools that interested me.	0.54 (0.56)	0.46
	5. I considered the pros and cons of my current school before enrolling.	0.63 (0.68)	0.55
	11. I am informing myself about the different possible directions of my future career.	0.54 (0.52)	0.39
	14. Before I enrolled in high school, I discussed with other people how capable I was of successfully attending it.	0.51 (0.60)	0.47
Factor 2: Exploration outside education profile	7. I have a gift for some vocations beside the one I am currently schooling for.	0.52 (0.55)	0.35
	9. I’m researching whether jobs outside of the profession I’m currently training for are suitable for me.	0.78 (0.76)	0.50
	13. I am interested in the possibilities of further education outside the profession which I am currently studying for.	0.40 (0.42)	0.30
Factor 3: Career commitment	2. I feel more satisfaction when I study material from vocational subjects than from other subjects.	0.64 (0.68)	0.63
	4. I can see myself with satisfaction doing the job I’m currently training for after completing my education.	0.75 (0.71)	0.64
	6. The knowledge I acquire from vocational subjects will help me a lot in my future job.	0.73 (0.76)	0.67
	8. I feel very proud when I acquire knowledge in vocational subjects.	0.65 (0.73)	0.67
	10. My current education is the best path towards the future I am planning to have.	0.75 (0.72)	0.66
	12. Other people can see my positive energy as I gain professional skills in practical classes.	0.53 (0.53)	0.49

Note: CITC = Corrected Item–Total Correlation. *p* < 0.001 for all standardized factor loadings (λ). λ′ = standardized factor loading in modified model; λ = standardized factor loading in initial model.

## Data Availability

The raw data supporting the conclusions of this article will be made available by the authors on request.
